# Outcome analysis of infrapatellar fat pad partial resection or preservation in patients with anterior cruciate ligament reconstruction

**DOI:** 10.1038/s41598-023-30933-0

**Published:** 2023-04-28

**Authors:** Yixin Wen, Feng Xu, Yang Liu, Kaining Zhi, Junfeng Tan, Yong Jiang, Minghui Li, Hui Zhang

**Affiliations:** 1 Department of Orthopaedics, Fifth Hospital in Wuhan, Wuhan, China; 2 Blood Transfusion Department, Wuhan Hankou Hospital, Wuhan, China

**Keywords:** Diseases, Medical research, Risk factors, Signs and symptoms

## Abstract

The infrapatellar fat pad (IPFP) is one of the structures surrounding the knee joint that obscures exposure in minimally arthroscopy anterior cruciate ligament reconstruction (ACLR). Most surgeons excise the partial fat pad for better exposure of the knee. However, whether removal of IPFP in ACLR remained inconclusive. The purpose of this study was to investigate clinical outcomes of IPFP preservation or resection in patients with primary hamstring-graft ACLR. A total of 104 patients were assigned to receive either IPFP-R (n = 55) or IPFP-P (n = 49). There were no significant preoperative differences between the two groups. The anterior knee pain (AKP) and the Knee Injury and Osteoarthritis Outcome Score (KOOS) in the two groups both recovered compared with those at baseline, but the IPFP-P group recovered more significantly at 3-, 6-, 12-month, and 3-, 6-month of follow-up, respectively. When assessing the KOOS subclasses using minimum perceptible clinical improvement (MPCI), patients with IPFP-R failed to make significant improvement at 3 months in the symptoms, pain and sports subsets of the KOOS. Knee-related complications were not significantly different between the two groups, while the resection group had a higher incidence. These results suggested that ACLR with primary hamstring grafts can achieve good effects whether performed with IPFP resection or preservation; however, the improvements in anterior knee pain and knee joint functions are better for the patients with IPFP preservation. Therefore, surgeons should avoid the resection of IPFP as much as possible while fully exposing the wild view to ensure the ACLR.

## Introduction

Anterior cruciate ligament (ACL) rupture is a common injury that can lead to recurrent instability and degenerative changes in the knee^1^. Arthroscopic reconstruction performed with hamstring grafts is a well-established surgical intervention with the intended benefits being pain relief and functional improvement. Although current reports have estimated that most patients are satisfied with their ACLR, a substantial number of patients’ daily life is still compromised by persistent postoperative knee pain and impaired functional outcomes^2–4^.

The infrapatellar fat pad (IPFP) is a fatty mass located between the inferior pole of the patellar and the tibial tubercle below the patellar ligament^5^. In the minimally invasive approach, this fatty tissue can obscure the surgical field or is caused damage during confirming the origin of the ACL on the femoral and tibial sides by arthroscopy, so its partial removal is sometimes performed for traditional ACLR exposure. However, some studies have pointed out that this tissue plays a role in the blood supply of the ACL, patella, and patellar tendon through the reticular geniculate artery^6,7^. In addition, it fills gaps inside the knee joint during joint movement and delivers synovial fluid to the joint surface^8^. In contrast, some other studies believed that the abnormal IPFP could produce various proinflammatory cytokines such as TNF-α, IL-6, and IL-8, and thus might play a detrimental role in anterior knee pain (AKP)^9–11^. Therefore, the function of the fat pad remains controversial.

Recently, one study suggested that partial resection of the IPFP during ACLR did not affect clinical outcomes including anterior knee pain^12^. However, the other study showed that after ACLR, a decrease in the thickness change ratio of the fat pad appeared to affect post-operative anterior knee pain^13^. Moreover, numerous studies have shown that the preservation of IPFP had a better postoperative outcome during total knee arthroplasty^14–17^. Although the above two studies have reported the effects of IPFP after ACL reconstruction, there are very few published data in the literature assessing clinical outcomes after ACLR in patients with differing perioperative IPEP preservation, and thus no consensus has been reached yet.

Hence, we conducted this study to determine the effect of infrapatellar fat pad resection on (1) anterior knee pain; (2) knee function score and subclasses; (3) knee activity indices; and (4) knee-related complications.

## Materials and methods

### Patient involvement

This was a retrospective study that collected data from all patients who were referred to the senior authors’ knee clinic for ACLR between 2018 and 2020, with a minimum 2-year follow-up. This retrospective observational study was approved by the Biomedical Research Ethics Committee of Fifth hospital in Wuhan, and written informed consent was obtained from all patients. All methods and procedures were performed in accordance with the relevant guidelines and regulations.

Patients were diagnosed as having a ruptured ACL through magnetic resonance imaging (MRI), and/or on a clinical basis if they had a positive Lachman test with a soft endpoint, a positive anterior drawer test (ADT), and a positive pivot shift test as assessed by the senior author. To avoid selection bias, the inclusion criteria of this study were that patients must only underwent hamstring grafts for unilateral primary ACL reconstruction, and there were no previous bone or ligament injuries or surgery of the knee joint.

The exclusion criteria included any graft choice other than hamstring (bone-patellar tendon-bone [BPTB], quadriceps tendon grafts), allograft ligaments, previous arthroscopy or ACL surgery of any knee, other knee diseases, previous knee bony operation, and other ligament procedures either in the past or during the current procedure.

### Surgical procedure

**Figure 1:**
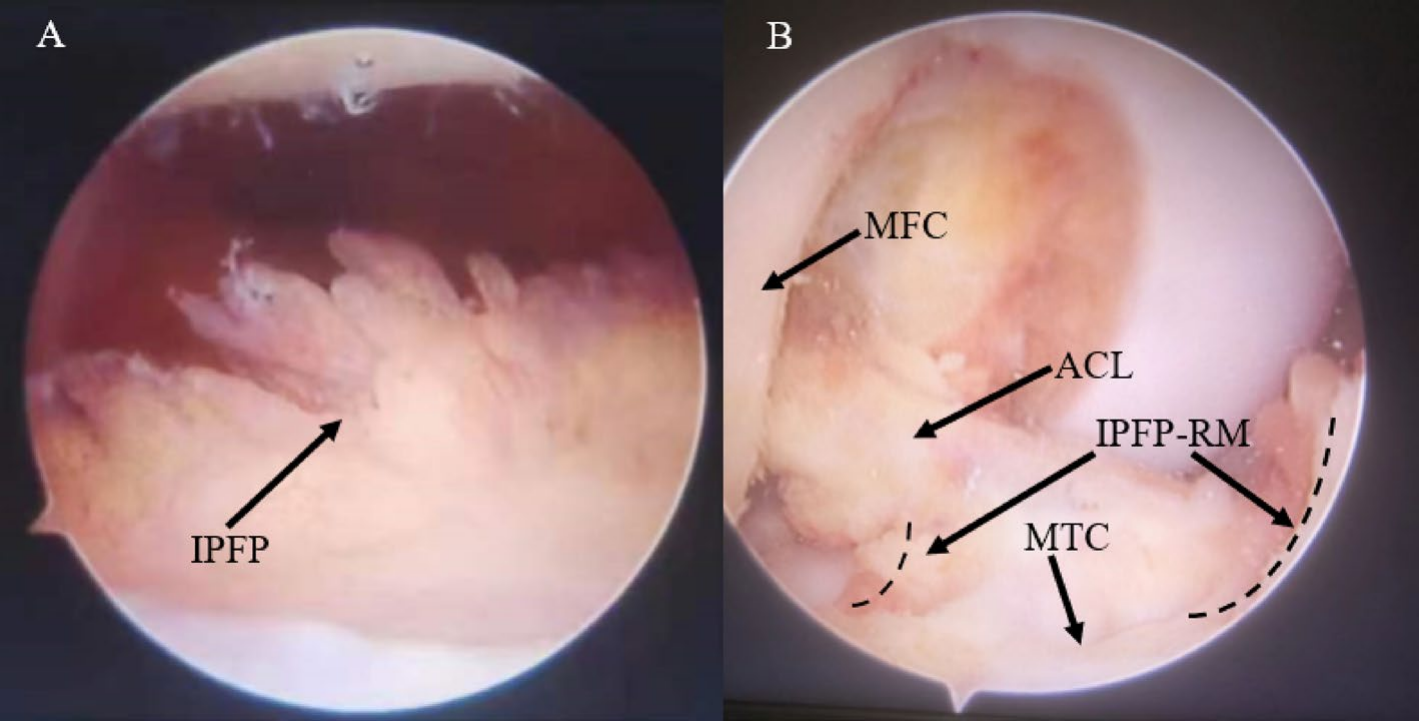
Representative intraoperative visualization in the infrapatellar fat pad resection group. (**A**) The intra-articular structure was obscured by infrapatellar fat pad under arthroscopy. (**B**) The extent of resection of infrapatellar fat (*IPFP* infrapatellar fat pad, *MFC* medial femoral condyle, *ACL* anterior cruciate ligament, *MTC* medial tibial condyle, *IPFP-RM* infrapatellar fat pad resection margin).

**Figure 2:**
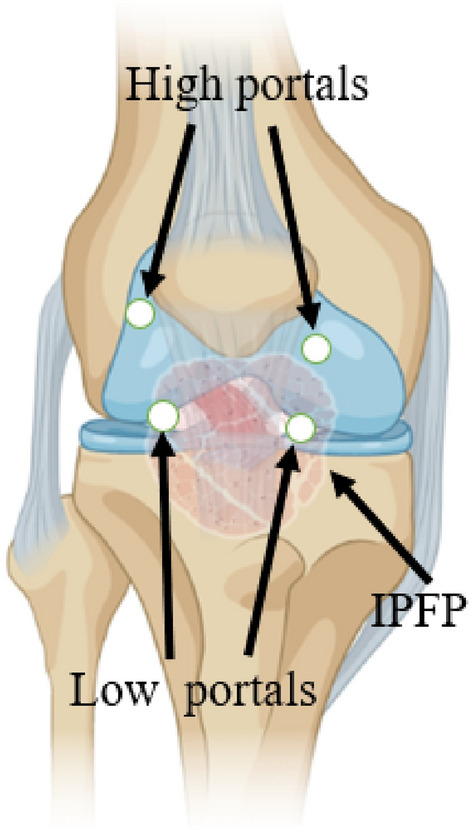
Schematic illustration of different portals in the infrapatellar fat pad preservation and resection group (*IPFP* infrapatellar fat pad).

All reconstruction procedures were performed by the same senior author. In the IPFP-R group, patients underwent only the medial border of the IPFP and intercondylar IPFP resection to better observe the tibial and femoral footprints of ACL (Fig. 1), especially the femoral footprints during deep knee flexion^18^. In the IPFP-P group, we performed a higher lateral portal, as previously reported by Sonnery-Cottet et al.^19^ (Fig. 2). The high approach was performed at the highest possible position near the lateral border of the patellar tendon and the inferior border of the patella. By adopting this position, the surgeon can avoid IPFP and gain an excellent wide view of the intercondylar notch and other intraarticular structures^19^. All patients followed our standard rehabilitation program after surgery and according to the patient’s responses, outpatient physiotherapy team regularly observed them for up to 24 months postoperatively.

### Clinical outcome assessment

We used the Anterior Knee Pain Scale as well as the Knee Injury and Osteoarthritis Outcome Score (KOOS) as our primary outcome measure^20^. This AKP scale required patients to answer a 13-item self-report questionnaire, which is called to measure the severity of symptoms during six activities considered to be particularly relevant to AKP syndrome, including walking, running, jumping, climbing stairs, squatting, and sitting for prolonged periods with the knee bent^20^. In addition, the KOOS subclasses (symptoms, pain, activities of daily living [ADL], sports, quality of life) and the Lysholm score were assessed as our secondary outcome. Moreover, as scarring of the fat pad may cause patellar down and limit the range of motion (ROM) after knee surgery^21^, we also measured ROM and some other clinical outcomes such as knee joint stability, patellar tendon tenderness, and pain with the half-squat test or single-leg hop test. These clinical outcomes between the groups were compared preoperatively and at 3-, 6-, 12-, and 24-month postoperative intervals. Any postoperative knee-related complications were also recorded and compared in addition to demographic data including age, gender, injury mechanism, and smoking status et al.

### Statistical analysis

The preoperative and postoperative scores of the 2 groups were compared using the unpaired t-test if the data were normally distributed or the Mann–Whitney U test if data were not normally distributed. Minimum perceptible clinical improvement (MPCI) was used to determine clinically significant improvements between preoperative and postoperative scores, and it was considered a clinically significant improvement when the MPCI in the KOOS and Lysholm scores was 10 points or more^22^. All statistical analyses were performed using SPSS Version 22.0 software and statistical significance was defined as *P* < 0.05.

### Ethics approval

This study was carried out in accordance with the recommendations of Ethics Committee of the Fifth hospital in Wuhan. All subjects gave written informed consent in accordance with the Declaration of Helsinki.

### Consent to participate

Informed consent was obtained from all individual participants included in the study.

### Consent for publication

Written consent to publish this article was obtained from study participants. Proof of consent to publish from study participants can be requested at any time.

## Results

A total of 152 patients underwent ACLR procedures during the study period; however, 18 of these had previous injuries in the ipsilateral knee joint and were therefore excluded from the study, leaving 134 patients who had only a primary hamstring graft ACLR. Twenty-four patients had missing follow-up data, and 6 patients with previous knee arthroscopy were also excluded from the study. Thus, 104 patients were available for final analysis.

Finally, there were 55 patients in the IPFP-R group and 49 patients in the IPFP-P group. Demographics are shown in Table 1; there were no significant differences between the 2 groups in basic data such as mean age, gender, BMI, causes, and sides of injury et al. (all *P* > 0.05).

**Table 1:** Demographics of patients in both groups.

Variable	IPFP-R group (n = 55)	IPFP-P group (n = 49)	*P* value
Age (years)	29.8 ± 9.3	27.5 ± 8.6	0.20
Gender (%)			
Male	40 (72.7%)	37 (75.5%)	0.75
Female	15 (27.3%)	12 (24.5%)	
Height (cm)	165.6 ± 9.9	163.6 ± 10.4	0.32
Weight (kg)	65.1 ± 14.6	62.9 ± 13.8	0.43
BMI (kg/m^2^)	24.3 ± 4.0	23.7 ± 3.5	0.42
Smoking (%)			0.97
Yes	21 (38.2%)	17 (34.7%)	
Quit > 6 months	7 (12.7%)	10 (20.4%)	
Never	27 (49.1%)	22 (44.9%)	
Side of injury (%)			0.95
Right	25 (45.5%)	22 (44.9%)	
Left	30 (54.5%)	27 (55.1%)	
Cause of injury (%)			0.90
Athletic injury	26 (47.3%)	24 (49.0%)	
Traffic accident	16 (29.1%)	13 (26.5%)	
Others	13 (23.6%)	12 (24.5%)	
Duration before surgery (days)	85.6 ± 74.2	94.3 ± 79.1	0.56

Values are expressed as mean ± standard deviation or n (%).

*IPFP* infrapatellar fat pad, *BMI* Body mass index, *IPFP-R* infrapatellar fat pad resection, *IPFP-P* infrapatellar fat pad preservation.

**Figure 3:**
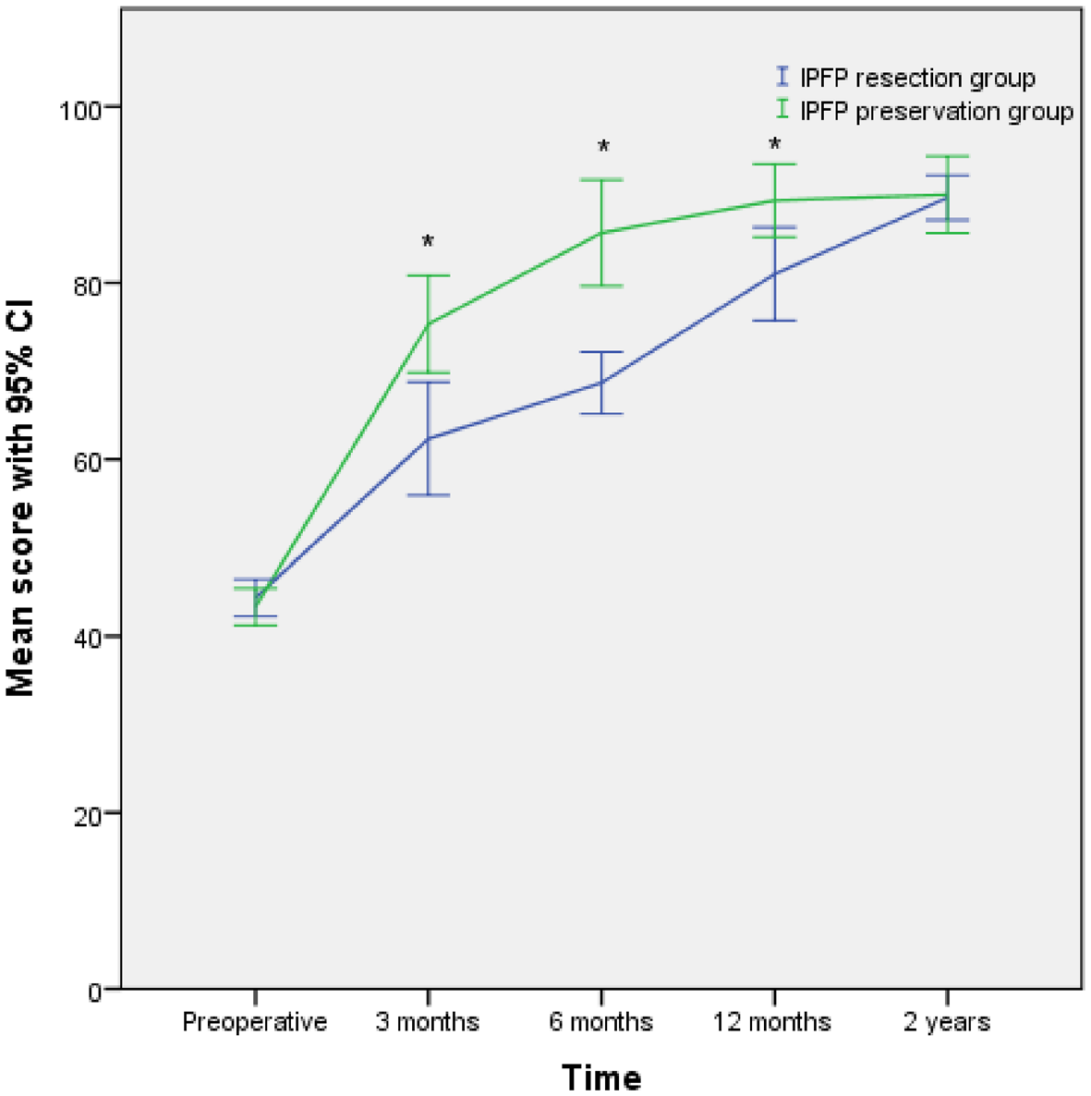
The mean overall Anterior Knee Pain Scale for both groups at different intervals with a 95% confidence interval (CI). *P < 0.05 between the 2 group scores at 3 months, 6 months, and 12 months postoperatively (*IPFP* infrapatellar fat pad).

**Figure 4:**
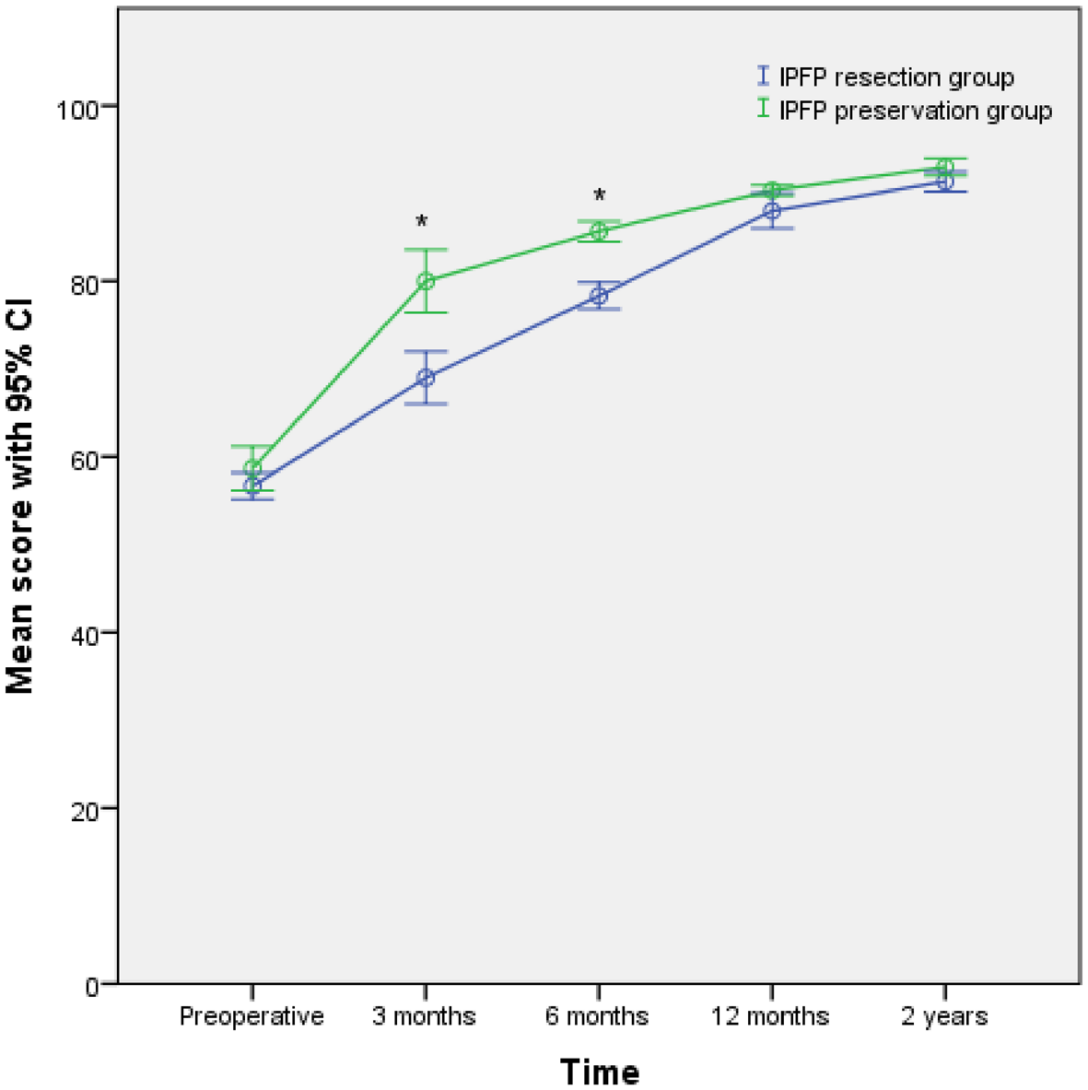
The mean overall KOOS for both groups at different intervals with a 95% confidence interval (CI). *P < 0.05 between the 2 group scores at 3 months and 6 months postoperatively (*IPFP* infrapatellar fat pad).

Preoperative AKP was found no difference in both groups (*P* = 0.68). After the operation, however, more significant AKP relief was found in the IPFP-P group than that in the IPFP-R group at 3 months, 6 months, and 12 months (*P* < 0.01, *P* = 0.01, and *P* = 0.03, respectively) (Fig. 3). As time went on, there was no significant difference in AKP between the two groups 2 years after surgery (*P* = 0.91). Similarly, when analyzing the KOOS, there was no significant difference between the two group scores preoperatively (both* P* > 0.05), and the two groups both had significant improvement after surgery, whereas there were significant differences between the two groups at 3 months and 6 months (*P* < 0.01 and *P* = 0.04) (Fig. 4).

**Figure 5:**
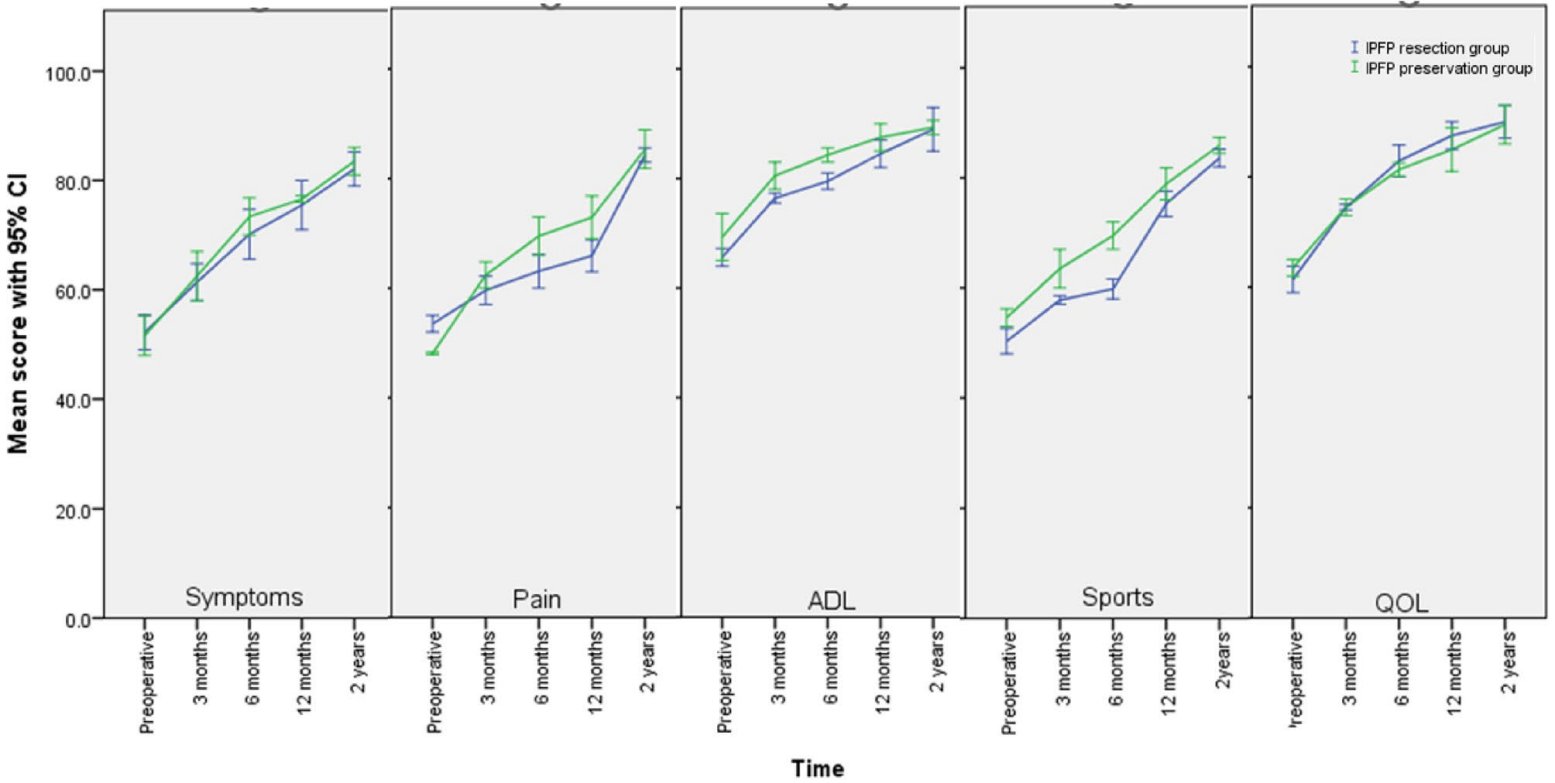
The KOOS subclass mean scores for both groups at different intervals with a 95% confidence interval (CI) (*IPFP* infrapatellar fat pad, *ADL* activities of daily living, *QOL* quality of life).

As for the KOOS subsets, there was no significant difference in the KOOS subclass scores between the 2 groups preoperatively or at any interval of the subsequent follow-up assessments (Fig. 5) (all *P* > 0.05). However, when assessing the mean MPCI between the two groups at different intervals (Table 2), we found that patients with IPFP resection had no significant clinical improvement in the 3-month mean symptom subset. Patients with IPFP resection also failed to make any clinically significant improvement in the mean pain and sports scores at the 3- and 6-month intervals, whereas no significant sports improvement at 3 months was also found in the patients with IPFP preservation. Except for these points, the both groups at all other follow-up evaluation intervals in all KOOS subsets showed significant clinical improvement. In the analysis of Lysholm score, compared with the mean preoperative scores, the patients with either preservation or resection both showed comparable clinically significant improvements in the postoperative scores (Table 2).

**Table 2:** Mean preoperative and postoperative scores with a mean change of scores from preoperative scores in both groups.

Variable	Group	Preoperative	3 months		6 months		12 months		2 years	
		Score	Score	Dif	Score	Dif	Score	Dif	Score	Dif
KOOS										
Symptoms	IPFP-R	52.2	61.4	9.2*	69.5	17.3	75.5	23.3	82.1	29.9
	IPFP-P	51.6	62.5	10.9	73.4	21.8	76.6	25.0	83.5	31.9
Pain	IPFP-R	53.5	59.6	6.1*	63.1	9.6*	65.9	12.4	84.3	30.8
	IPFP-P	48.1	62.4	14.3	69.5	21.4	72.9	24.8	85.1	37.0
ADL	IPFP-R	65.6	76.4	10.8	79.5	13.9	84.5	18.9	88.9	23.3
	IPFP-P	69.3	80.5	11.2	84.3	15.0	87.5	18.2	89.3	20.0
Sports	IPFP-R	50.3	57.8	7.5*	59.8	9.5*	75.3	25.0	83.6	33.3
	IPFP-P	54.6	63.5	8.9*	69.5	14.9	78.9	24.3	85.9	31.3
QOL	IPFP-R	61.4	74.5	13.1	82.9	21.5	87.5	26.1	90.0	28.6
	IPFP-P	63.5	74.5	11.0	81.6	18.1	88.3	24.8	89.5	26.0
Lysholm	IPFP-R	59.8	70.2	10.4	74.5	14.6	79.0	19.2	88.5	28.7
	IPFP-P	61.7	77.9	16.2	83.6	21.9	87.1	25.4	89.6	27.9

Dif is a change of score from the preoperative score.

No clinical improvement from the preoperative score.

*IPFP-R* infrapatellar fat pad resection, *IPFP-P* infrapatellar fat pad preservation, *KOOS* knee injury, and osteoarthritis outcome score, *ADL* activities of daily living, *BMI* body mass index, *QOL* quality of life.

Preoperative and postoperative intergroup and intragroup comparisons of knee joint activity between the two groups are summarized in Table 3. The ROM limitation, stability, patellar tendon tenderness, and pain with the half-squat test or single-leg hop test all had a significant improvement in both groups than before surgery, but during the 3-month follow-up periods, fewer patients who experienced significant tenderness in the patellar tendon were observed in the IPFP-P group than in the IPFP-R group (9.1% vs 27.3%). However, at 12 months and 2 years, there was a significant recovery in the IPFP-R group and the intergroup difference was not significant (both* P* = 0.75). In addition to this point, no other significant differences were found between the two groups at either preoperative or any postoperative follow-up intervals.

**Table 3:** Intergroup and intragroup comparison of knee joint activity before and after surgery in both groups.

Variable	Group	Preoperative	3 months	6 months	12 months	2 years	*P* value*
ROM limitation	IPFP-R	42 (76.4%)	3 (5.5%)	3 (5.5%)	1 (1.8%)	0	< 0.01
	IPFP-P	39 (79.6%)	2 (4.1%)	2 (4.1%)	0	0	< 0.01
	*P* value	0.70	0.75	0.75	0.94	1.0	
Stability by ADT test	IPFP-R	35 (63.6%)	2 (3.6%)	2 (3.6%)	0	0	< 0.01
	IPFP-P	34 (69.4%)	2 (4.1%)	2 (4.1%)	0	0	< 0.01
	*P* value	0.54	0.91	0.91	1.0	1.0	
Stability by Lachman test	IPFP-R	30 (54.5%)	3 (5.5%)	2 (3.6%)	0	0	< 0.01
	IPFP-P	28 (57.1%)	2 (4.1%)	2 (4.1%)	0	0	< 0.01
	*P* value	0.79	0.75	0.91	1.0	1.0	
Patellar tendon tenderness	IPFP-R	45 (81.8%)	15 (27.3%)	9 (16.4%)	3 (5.5%)	3 (5.5%)	< 0.01
	IPFP-P	45 (91.8%)	5 (9.1%)	4 (8.2%)	2 (4.1%)	2 (4.1%)	< 0.01
	*P* value	0.14	0.03	0.21	0.75	0.75	
Half-squat test	IPFP-R	29 (52.7%)	16 (29.1%)	10 (18.2%)	5 (9.1%)	2 (3.6%)	0.01
	IPFP-P	30 (61.2%)	10 (20.4%)	7 (14.3%)	3 (6.1%)	3 (10.2%)	< 0.01
	*P* value	0.39	0.31	0.60	0.58	0.56	
Single-leg hop test	IPFP-R	31 (56.4%)	19 (34.5%)	12 (21.8%)	6 (10.9%)	1 (1.8%)	0.02
	IPFP-P	33 (67.3%)	17 (34.7%)	10 (20.4%)	6 (12.2)	1 (2.0%)	0.12
	*P* value	0.25	0.99	0.86	0.83	0.94	

Values are expressed as n (%).

Range of motion (ROM) limitation was defined as less than 10° of knee extension or flexion compared to the healthy side.

*IPFP-R* infrapatellar fat pad resection, *IPFP-P* infrapatellar fat pad preservation, *ROM* range of motion, *ADT* anterior drawer test.

*The *P* value was obtained from the comparison of preoperative and 3 months after surgery.

Complications in the two groups are shown in Table 4 (*P* = 0.83). There were 6 patients who had complications postoperatively in both groups, respectively. A total of 7 patients were related to infection, 3 patients were in the IPFP-R group and the remaining 4 patients were in the IPFP-P group. The four patients with deep infection were subsequently given multiple knee joint washes and intravenous antibiotics, while the patient with superficial wound infection only underwent several wound dressing changes, and satisfactorily these patients all recovered. Moreover, we observed no metal work irritation, avascular necrosis, or graft failure in either group during the study. Additionally, in the IPFP-R group, two of these complications were postoperative venous thrombosis in lower extremities and one was related to patellar fracture. Similarly, in the IPFP-P group, one patient also developed venous thrombosis in the lower extremities, and one patient developed a patella fracture. Effectively, these patients all had significant improvements through a series of treatments.

**Table 4:** Complications in both groups.

Complication	IPFP-R group (n = 6)	IPFP-P group (n = 6)
Infection		
Superficial	2 (33.3%) (both the fourth day postoperatively)	2 (33.3%) (the third and fourth day postoperatively)
Deep	1 (16.7%) (the second day postoperatively)	2 (33.3%) (the third and fifth day postoperatively)
Graft failure	0	0
Metal work irritation	0	0
Venous thrombosis in lower extremities	2 (33.3%) (the third and fourth day postoperatively)	1 (16.7%) (the third day postoperatively)
Patellar fracture	1 (16.7%) (the 8-month postoperatively)	1 (16.7%) (the 15-month postoperatively)
Avascular necrosis	0	0

Values are expressed as n (%).

*IPFP-R* infrapatellar fat pad resection, *IPFP-P* infrapatellar fat pad preservation.

## Discussion

The infrapatellar fat pad, as a cushion between the patellar tendon and the anterior tibial plateau, can mask exposure to the surgical area in a knee arthroscopy operation. Resection of the fat pad enhances exposure, especially in knee arthroplasty, but there are still some controversies that its resection on the effect on clinical outcomes. Similarly, whether resection of the fat pad in ACLR remained inconclusive and urged to be further investigated. Therefore, we sought to determine the effect of fat pad resection on clinical outcomes in ACLR.

Anterior knee pain was the most frequently reported outcome measure, with many studies providing data on its association with IPFP resection^15,23–25^. Tanaka et al.^15^ found that patients with rheumatoid arthritis who underwent IPFP resection had a higher incidence of AKP at the follow-up of 1 to 2 months and the relationship persisted at 28 to 38 months follow-up, with pain occuring in 43% of resection patients, while with only 4% in the preservation patients. An increase in pain following resection of the IPFP was similarly seen by Pinsornsak et al.^23^. In their study, there were significant differences in the incidence of AKP between the resection and preservation of fat pad groups at 3-, 6-, and 12-month follow-up periods (all *P* < 0.05), respectively^23^. On the contrary, using Visual Analogue Scale pain scores, Seo et al.^25^ found there was no difference for the patients who underwent the IPFP resection or preserved during the first 72 h following the total knee arthroplasty. Our study, however, showed that IPFP resection did adversely impact the postoperative AKP within one year after ACLR by Anterior Knee Pain Scale. In addition, when assessing these findings in more detail for the KOOS subtype of pain, the IPFP resection group still failed to make an MPCI at the 3- and 6-month intervals. We think it may be the reason that branches of the femoral, saphenous, obturator, and sciatic nerves pass through IPFP so that IPFP plays an important role in pain perception^26^. Moreover, in the present study, the IPFP of patients in the resection group was partially resected, and a decrease in the threshold of pain sensation may result from an increase in free nerve endings^13^. In addition, previous animal model studies have confirmed that when the rat experienced ACLR, trauma or patellar tendinopathy, free nerve endings in the IPFP used to be accompanied by fibrosis, vascularity, and other histopathological changes^27,28^. All in all, the results showed that IPFP resection did significantly influence postoperative AKP in patients with ACLR.

Another main finding of this study was that IPFP resection was associated with KOOS after ACLR. However, when assessing the finding in more detailed KOOS subclasses, both resection and preservation groups failed to make an MPCI at some postoperative intervals. Patients with IPFP resection made no significant improvement at 3 months in the symptoms, pain, and sports subsets of the KOOS. It could be that IPFP is thought to play a biomechanical role in adjusting the pressure of the patellofemoral joint and the anterior chamber in the knee joint^29^, while the resection of IPFP could cause AKP, which leads to higher pressure in the knee joint, thereby decreasing the function. At the same time, the reason also further explains that patients after IPFP resection with postoperative pain were often accompanied by worse symptoms and sports. Additionally, we found that patients with IPFP preservation also failed to improve in the sports subset at a 3-month follow-up. The reason why there is no obvious improvement at this stage may be that the patients with ACLR are in the rehabilitation phase within 3 months, during this phase it is true that the patients will experience some physical pain, swelling and limited range of motion, so sports are often limited in this early stage^22^. However, the reason cannot deny the fact that the resection of IPFP indeed affects the postoperative recovery of knee joint function.

For range of motion (ROM) limitation, knee joint stability, patellar tendon tenderness, and pain with the half-squat test or single-leg hop test, both groups had a significant improvement than before surgery. However, although there was no statistical difference between the two groups on all parameters at different follow-up intervals, except for patellar tendon tenderness at 3 months postoperatively (both P < 0.05), there was a higher incidence of positivity in the IPFP resection group than the IPFP preservation group. At this point, our findings, therefore, supported findings from a previous study^12^, which similarly indicated that there were no statistical differences in the clinical assessment parameters between the IPFP preservation and resection groups, including ROM limitation, half squat, and single-leg hop test. However, the difference in the patellar tendon tenderness may be that, on the one hand, the above-mentioned decrease in pain threshold is due to increased free nerve endings as a result of abnormal IPFP^13^, and on the other hand, the theory that intra-articular fibrosis and scarring after excision of the fat pad leads to shortening of the patellar tendon^30^, as a previous study demonstrated that the patellar tendon shortening occurred in patients with resection of IPFP but not in patients with the IPFP preservation during surgery^14^. So, further studies on the morphology difference of the IPFP further need to be investigated.

Additionally, we evaluated complications in both groups after ACLR. In both groups, we found infections and venous thrombosis in the lower extremities to be the most common complications. An anatomic vascular study showed that Patellar bone vascularity might not be affected by disruption of infrapatellar blood supply^31^. Our study showed that there was no evidence of avascular necrosis and graft failure in patients with IPFP resection, which is consistent with that reported in the literature. Although 6 patients in each of the two groups induced complications, we found a higher incidence of complications in patients undergoing IPFP resection (12.2% vs 10.9%). As a result, we believe that the significance of IPFP preservation for these patients with ACLR may be valuable.

This study had limitations that should be acknowledged. First, Our patients’ postoperative MRI data cannot be obtained persistently, and thus, we were unable to utilize imaging as an result measure, which may have led to an underestimation of the IPFP change ratio on the outcome effect^13,32^. Second, AKP is a subjective measurement, which may determine the accuracy and reliability of the outcomes^33^. Third, no pre- and post-operative psychological data were obtained, which could be useful for the potential influence^34,35^. Despite these shortcomings, our study showed several significant findings that confirm the significance of IPFP preservation for patients with ACLR. A larger prospective study should be conducted in more detail to better assess the importance of IPFP preservation in these patients.

## Conclusions

Anterior cruciate ligament reconstruction with primary hamstring grafts can achieve good effects whether performed with IPFP resection or preservation; however, the improvements in patients’ anterior knee pain and knee joint functions are better in the preservation group. Therefore, the ACLR with IPFP preservation should be advocated, and surgeons should avoid the resection of IPFP as much as possible while fully exposing the wild view to ensure the ACLR.

## Data Availability

The data and code used to support the findings of this study are available from the corresponding author upon request.
